# Positive Mental Health and Well-Being among a Third Level Student Population

**DOI:** 10.1371/journal.pone.0074921

**Published:** 2013-08-29

**Authors:** Martin P. Davoren, Eimear Fitzgerald, Frances Shiely, Ivan J. Perry

**Affiliations:** Department of Epidemiology and Public Health, University College Cork, Cork, Ireland; Federal University of Rio de Janeiro, Brazil

## Abstract

**Introduction:**

Much research on the health and well-being of third level students is focused on poor mental health leading to a dearth of information on positive mental health and well-being. Recently, the Warwick Edinburgh Mental Well-being scale (WEMWBS) was developed as a measurement of positive mental health and well-being. The aim of this research is to investigate the distribution and determinants of positive mental health and well-being in a large, broadly representative sample of third level students using WEMWBS.

**Methods:**

Undergraduate students from one large third level institution were sampled using probability proportional to size sampling. Questionnaires were distributed to students attending lectures in the randomly selected degrees. A total of 2,332 self-completed questionnaires were obtained, yielding a response rate of 51% based on students registered to relevant modules and 84% based on attendance. One-way ANOVAs and multivariate logistic regression were utilised to investigate factors associated with positive mental health and well-being.

**Results:**

The sample was predominantly female (62.66%), in first year (46.9%) and living in their parents’ house (42.4%) or in a rented house or flat (40.8%). In multivariate analysis adjusted for age and stratified by gender, no significant differences in WEMWBS score were observed by area of study, alcohol, smoking or drug use. WEMWBS scores were higher among male students with low levels of physical activity (p=0.04). Men and women reporting one or more sexual partners (p<0.001) were also more likely to report above average mental health and well-being.

**Conclusion:**

This is the first study to examine positive mental health and well-being scores in a third level student sample using WEMWBS. The findings suggest that students with a relatively adverse health and lifestyle profile have higher than average mental health and well-being. To confirm these results, this work needs to be replicated across other third level institutions.

## Introduction

Suicide, deliberate self-harm and the negative aspects of mental health and well-being have previously been the primary focus of much research. Studies from around the world focusing on the mental health and well-being of third level students have investigated risky behaviours and poor mental health and well-being [1–6]. Much of this research has highlighted alcohol and drug abuse, financial pressures and academic concerns [1,2] as determinants of mental health and well-being. However, a dearth of information on positive mental health and well-being among this population has been identified.

Historically, humankind has always been fascinated with the determinants of human health, happiness and well-being [7]. Positive mental health can be defined as “the scientific study of those positive strengths and virtues that enable people & communities to reach optimal levels of health, happiness and well-being” [8]. This adopts the view that positive mental health and well-being is a positive concept which goes beyond healthy lifestyles to overall well-being and replaces the traditional medical perspective which has mainly focused on the treatment of mental illness [9]. As highlighted by Seligman, positive mental health and well-being is grounded in the philosophy of positive psychology [8,10] and health promotion [11].

Recently, the Warwick Edinburgh Mental Well-Being Scale (WEMWBS) was developed as a measurement of positive mental health and well-being. In 2007, a report on its validity, variability and social desirability among a general and student population was published [12]. However, the determinants of positive mental health and well-being have yet to be investigated among a third level student population utilising WEMWBS. Thus, the aim of this research is to investigate the distribution and determinants of positive mental health and well-being in a large, broadly representative sample of Irish third level students.

## Materials and Methods

### Sampling & Participants

Undergraduate students registered to degree programmes at one large third level institution were randomly sampled using probability proportional to size (PPS) sampling. The primary focus of this research was on hazardous alcohol consumption. The required sample size of 2,686 students was calculated based on a prevalence of hazardous alcohol consumption of approximately 70% with a precision of 1.5%. Lecturers or module coordinators were contacted to request permission to distribute and collect questionnaires during fifteen minutes of lecture time on a date convenient to them between March 12^th^ and 23^rd^, 2012. Students were initially briefed on the aims and objectives of the study along with its confidential, anonymous and voluntary nature. Questionnaires were then distributed and students given fifteen minutes for completion. All questionnaires were immediately collected and a post-questionnaire information sheet distributed. As an incentive, all participants were invited to enter a draw to win a tablet computer following survey completion. As questionnaire completion was anonymous, each student was advised to send an e-mail to the lead author with his or her name and e-mail address to enter the prize draw.

In total, 2,332 students completed this face-to-face lecture theatre based, cross-sectional survey. The response rate for those registered to the specific modules visited was 51% but for those in attendance an 84% response rate was achieved. Of these, 2,044 undergraduates completed the full fourteen questions involved in the Warwick-Edinburgh Mental Wellbeing Scale and are the subject of this analysis. Age, gender and the course profiles of the sample collected was similar to those registered with the university. The sample is representative of undergraduate men and women when compared to the specific institution demographics with more women (56%) registered to undergraduate degrees than men (44%). Furthermore, it was representative of university faculty. The institution reports 33% of undergraduate students were registered to the faculty of Arts, 21% to Law and Business, 27% to Science and Engineering while 19% were registered to Health & Welfare courses in 2011-12.

### Questionnaire

The questionnaire was based on previous third level surveys conducted in Ireland [3] and it addressed a range of topics including mental health and well-being, alcohol use, smoking, drug use, physical activity, sexual health, diet and self-reported height and weight. Positive mental health and well-being was estimated using the Warwick-Edinburgh Mental Well-being Scale (WEMWBS) [12]. The Cronbach’s Alpha measure of internal consistency for WEMWBS in this study was 0.90.

The questionnaire also included additional validated instruments such as the Alcohol Use Disorders Identification Test for Consumption (AUDIT-C) and the International Physical Activity Questionnaire (IPAQ) [13]. The Clinical Research Ethics Committee, University College Cork, Ireland, granted ethical approval for this research.

### Data management & Statistical analysis

Data were scanned, checked and verified using TeleForm TM scanning processes. The estimated error rate for data entry was 0.06% based on manual checking of a 10% sample of all scanned questionnaires. WEMWBS, AUDIT-C and IPAQ scores were computed in accordance with instrument guidelines. WEMWBS scores were divided into categories of mental well-being as defined by Braunholtz et al [14]. Below average mental wellbeing was defined as a WEMWBS score of more than one standard deviation below the mean, average mental wellbeing was within one standard deviation of the mean and above average mental wellbeing was over one standard deviation above the mean. BMI was estimated from self-reported height and weight. Data were analysed using IBM SPSS Statistics 20. One-way ANOVAs, univariate and multivariate logistic regression analysis were utilised to investigate factors associated with above average mental health and well-being. Multivariate logistic regression, applying backward elimination, was undertaken to investigate the combination of risk factors associated with above average mental health and well-being. Risk factors appearing as statistically significant (P < 0.20) in the univariate logistic regressions were used to obtain an initial multivariable logistic regression fit.

## Results

Baseline characteristics of the student sample are presented by gender in Table 1. Faculty, year in college and accommodation differed significantly by gender (p<0.05). As expected, women were more likely to be taking Health and Welfare undergraduate degrees. Over 40% of the sample were in first year. Gender distribution varied slightly by college year with a higher proportion of women in second year. Men were less likely to be living in campus accommodation (8.93% vs. 14.9%) and more men reported living at home with their parents than women (47.74% vs. 39.2%).

**Table 1:** Characteristics of students sociodemographic factors and WEMWBS scores by gender [N (%)/Mean (SD)].

		**Males (N=755)**	**Females (N=1,267)**	**Chi-Square**
**Age**	<=18	96 (12.9%)	163 (13.2%)	0.077
	19	248 (33.3%)	382 (30.9%)	
	20	145 (19.5%)	278 (22.5%)	
	21	93 (12.5%)	189 (15.3%)	
	22+	162 (21.8%)	225 (18.2%)	
**Faculty**	Science/Engineering	235 (31.4%)	275 (21.9%)	**<0.001**
	Arts/Social Science/Education	268 (35.8%)	501 (40%)	
	Law & Business	174 (23.3%)	234 (18.7%)	
	Health & Welfare	57 (7.6%)	228 (18.2%)	
	Other	14 (1.9%)	16 (1.3%)	
**College Year**	First	400 (53.1%)	548 (43.3%)	**<0.001**
	Second	178 (23.6%)	379 (29.9%)	
	Third	130 (17.2%)	239 (18.9%)	
	Fourth	46 (6.1%)	101 (8.0%)	
**Accommodation**	Campus Accommodation	67 (8.93%)	188 (14.9%)	**<0.001**
	Rented House/Flat	289 (38.53%)	531 (42.2%)	
	Parents’ House	358 (47.74%)	494 (39.2%)	
	House Owner	36 (4.8%)	46 (3.7%)	
**Physical Activity**	Low	216 (29.2%)	386 (31%)	0.693
	Moderate	324 (43.8%)	529 (42.5%)	
	High	200 (27%)	330 (26.5%)	
**Smoking**	Yes	201 (27%)	320 (25.6%)	0.5
	No	544 (73%)	929 (74.4%)	
**Alcohol Consumption**	Hazardous consumption	492 (65.2%)	855 (67.5%)	0.285
	Non-hazardous consumption	263 (34.8%)	412 (32.5%)	
**Drug use**	Yes	305 (40.4%)	334 (26.4%)	**<0.001**
	No	450 (59.6%)	933 (73.6%)	
**No. of sexual partners**	None	168 (22.8%)	237 (19.8%)	**<0.001**
	1-3	324 (44%)	623 (51.9%)	
	4-5	79 (10.7%)	177 (14.8%)	
	6+	166 (22.5%)	163 (13.6%)	
**Mental Health & Well-being**	Below average	91 (12.1%)	182 (14.4%)	0.311
	Average	545 (72.3%)	901 (71.1%)	
	Above average	118 (15.6%)	184 (14.5%)	
	WEMWBS mean Score	50.2 (8.4)	49.3 (8.4)	**0.015**

No statistically significant differences were observed between men and women in relation to age, physical activity, alcohol consumption or smoking. Over 40% of students reported moderate levels of physical activity, while in excess of 65% of students reported hazardous alcohol consumption. Over a quarter of students reported having smoked 100 cigarettes in their lives. Men were significantly more likely to report six or more sexual partners compared to women (22.5% vs. 13.6%; p<0.001). In addition, men were more likely to report taking at least one form of illicit drug use during the previous 12 months (40.4% vs. 26.4%; p<0.001).

**Figure 1:**
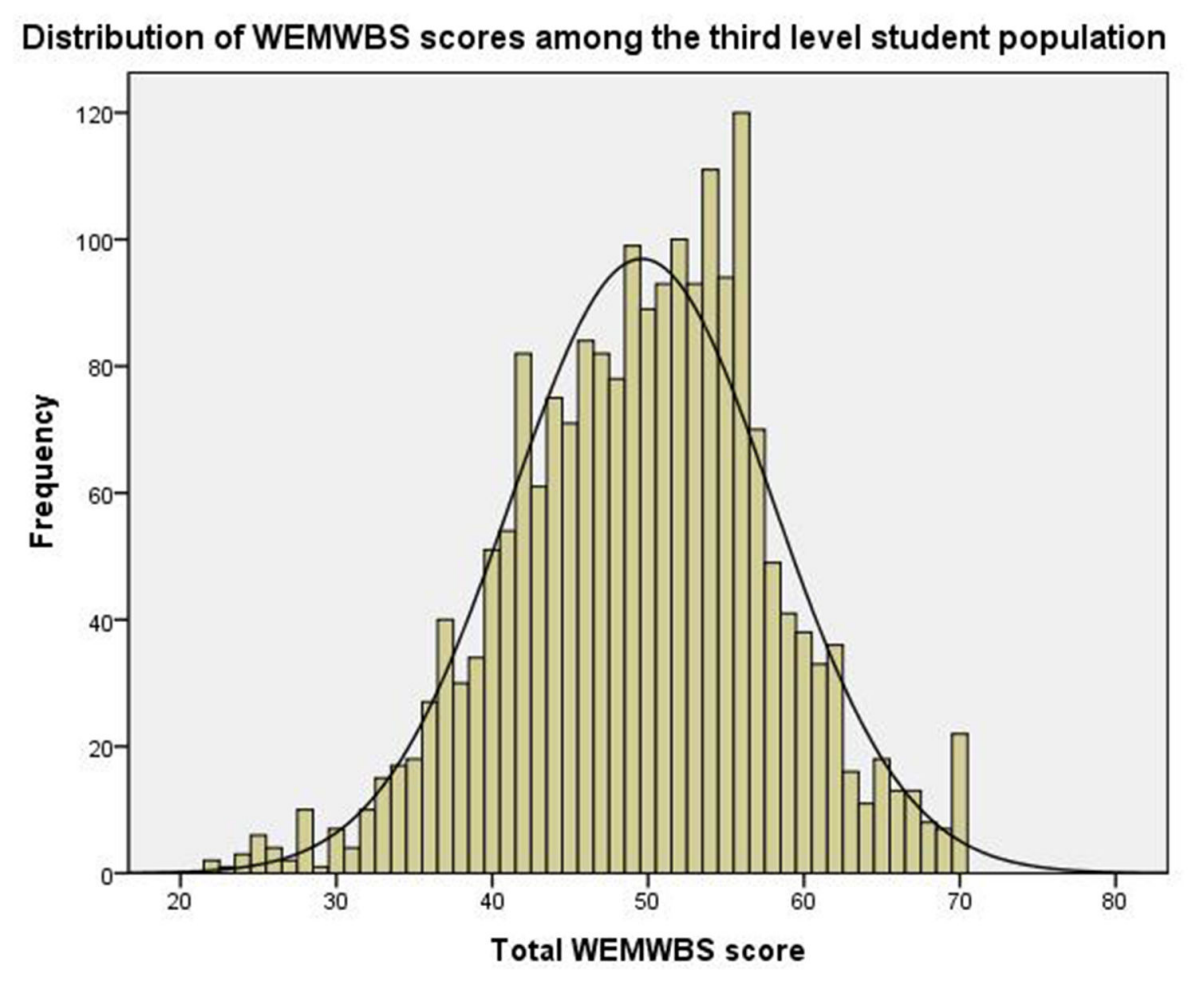
Distribution curve for WEMWBS scores among a third level student population

WEMWBS scores were normally distributed in this population (Figure 1). Mean WEMWBS scores were slightly higher in men than in women (p=0.015). Distributions of men and women’s WEMWBS scores, categorised as below average, average and above average mental well-being, are displayed in Table 1.

Analysis of variance was undertaken to investigate whether significant differences in WEMWBS scores were evident among different socio-demographic, social and lifestyle factors reported by students. Analysis was conducted separately for men and women (Table 2).

**Table 2:** WEMWBS scores by Gender & Socio-demographic factors.

			**Males**		**Females**	
			**Mean WEMWBS score (95% CI)**	**p-value (ANOVA)**	**Mean WEMWBS score (95% CI)**	**p-value (ANOVA)**
**Socio-demographic Indicators**	**Age group**	**= < 18**	49.58 (48.07-51.1)	p=0.45	49.4 (48.17-50.63)	**p=0.04**
		**19**	50.56 (49.53-51.6)		48.93 (48.11-49.75)	
		**20**	50.55 (49.05-52.04)		48.46 (47.47-49.45)	
		**21**	51.17 (49.71-52.64)		49.52 (48.27-50.78)	
		**22+**	49.46 (48.08-50.85)		50.7 (49.6-51.8)	
	**Year of study**	**1^st^ year**	49.91 (49.11-50.7)	**p=0.012**	49.5 (48.81-50.2)	p=0.13
		**2^nd^ year**	49.39 (48.07-50.71)		48.89 (48.04-49.73)	
		**3^rd^ year**	52.36 (50.96-53.77)		48.74 (47.64-49.84)	
		**4^th^ year**	50.2 (49.62-50.82)		50.83 (49.2-52.46)	
	**Area of study**	**Science/Engineering**	50.34 (49.26-51.41)	p=0.44	49.32 (48.27-50.38)	p=0.32
		**Arts/ Social Science/ Education**	49.61 (48.63-50.58)		48.87 (48.13-49.6)	
		**Law/Business**	50.61 (49.31-51.9)		49.65 (48.58-50.72)	
		**Health & Welfare**	51.33 (49.13-53.54)		49.83 (48.81-50.86)	
		**Other**	52.36 (47.19-57.52)		46.25 (40.83-51.67)	
	**Accommodation**	**House Owner**	47.58 (45.41-49.76)	p=0.15	48.85 (46.28-51.41)	p=0.52
		**Parents House**	50.07 (49.17-50.98)		49.26 (48.51-50.01)	
		**Rented House**	50.45 (49.49-51.42)		49.05 (48.34-49.76)	
		**Campus Accommodation**	51.45 (49.65-53.25)		50.09 (48.86-51.31)	
**Lifestyle indicators**	**Smoking Status**	**Smoker**	50.27 (49.08-51.46)	p=0.89	48.54 (47.56-49.52)	p=0.06
		**Non -smoker**	50.18 (49.47-50.88)		49.59 (49.06-50.12)	
	**Drug Use-Ever**	**Yes**	50.16 (49.18-51.13)	p=0.86	49.23 (48.26-50.2)	p=0.9
		**No**	50.27 (49.51-51.03)		49.3 (48.77-49.83)	
	**Alcohol Use**	**Hazardous**	51.07 (50.36-51.77)	**p<0.001**	49.37 (48.82-49.93)	p=0.58
		**Non-Hazardous**	48.65 (47.56-49.73)		49.09 (48.25-49.94)	
	**Physical Activity**	**Low**	51.53 (50.39-52.66)	**p=0.013**	49.02 (48.17-49.87)	p=0.76
		**Moderate**	49.37 (48.46-50.29)		49.43 (48.73-50.14)	
		**High**	50.07 (49.58-50.8)		49.29 (48.39-50.19)	
	**BMI**	**Underweight**	49.14 (45.47-52.81)	p=0.89	48.79 (47.2-50.38)	p=0.76
		**Normal Weight**	50.12 (49.38-50.85)		49.4 (48.8-50.0)	
		**Overweight**	50.47 (49.00-51.93)		48.82 (47.46-50.19)	
		**Obese**	50.6 (49.67-53.53)		49.95 (46.94-52.96)	
	**No. of sexual partners**	**None**	48.77 (47.52-50.02)	p=0.07	48.15 (47.13-49.17)	p=0.17
		**1-3**	50.69 (49.8-51.57)		49.35 (48.7-49.99)	
		**4-5**	51.05 (49.22-52.88)		49.81 (48.56-51.06)	
		**6 or more**	50.51 (49.09-51.94)		49.43 (47.97-50.89)	

Among men, mean WEMWBS scores were similar in relation to age group, area of study, accommodation, smoking status, drug use, BMI and number of sexual partners. Mean WEMWBS scores differed significantly by year of study, alcohol use and physical activity. Third year students reported higher average WEMWBS scores than their first year (52.36 vs. 49.91; p=0.012), second year (52.36 vs. 49.39; p=0.012) and fourth year (52.36 vs. 50.2; p=0.012) counterparts. Mean WEMWBS scores were higher among hazardous alcohol consumers versus non-hazardous consumers (51.07 vs. 48.65: p<0.001). Evidence of a non-linear relationship between physical activity and mental health and well-being was recorded. Men reporting moderate physical activity have significantly lower WEMWBS scores than men reporting low levels of physical activity (49.37 vs. 51.53; p=0.013)

Among women, mean WEMWBS scores differed by age with those aged 22 and older reporting better mental health and well-being than their female counterparts aged 20. However, there was no difference in WEMWBS scores for lifestyle or socio-demographic characteristics among women.

### Logistic regression

Univariate logistic regression, stratified by gender, was undertaken to uncover risk factors associated with above average mental health and well-being. Among men, those who reported hazardous alcohol consumption and one or more lifetime sexual partners were more likely to report above average mental health and well-being. Those reporting low or moderate levels of physical activity were less likely to report above average mental health and well-being. Among women those reporting 1-3 or 6 or more lifetime sexual partners were more likely to report above average mental health and well-being as were those reporting illegal drug use. Results of univariate regression can be seen in Table 3. Covariates with a p-value less than 0.2 were included in the multivariable model and included illegal drug use, level of physical activity, alcohol use, BMI and number of lifetime sexual partners for men and year of study, illegal drug use and number of sexual partners for women.

**Table 3:** Univariate Logistic Regression: Risk factors associated with above average mental health and well-being.

		**Males**			**Females**		
		**OR**	**95%CI**	**p-value**	**OR**	**95%CI**	**p-value**
**Age group**	**= < 18**	1.00		**0.06**	1.00		0.31
	**19**	2.82	1.22-6.49		0.86	0.51-1.49	
	**20**	3.29	1.38-7.83		0.88	0.5-1.54	
	**21**	1.89	0.71-5.02		1.24	0.69-2.22	
	**22+**	2.1	0.87-5.11		1.32	0.75-2.3	
**Year of study**	**1^st^ year**	1.00		0.33	1.00		**0.16**
	**2^nd^ year**	1.16	0.71-1.91		0.88	0.6-1.28	
	**3^rd^ year**	1.57	0.94-2.63		0.86	0.55-1.34	
	**4^th^ year**	1.53	0.7-3.34		1.61	0.95-2.72	
**Area of study**	**Science/Engineering**	1.00		0.31	1.00		0.99
	**Arts/ Social Science/ Education**	0.68	0.41-1.13		0.92	0.61-1.39	
	**Law/Business**	1.13	0.67-1.88		0.91	0.57-1.49	
	**Health & Welfare**	1.2	0.57-2.52		0.97	0.6-1.59	
	**Other**	1.37	0.37-5.14		0.79	0.17-3.62	
**Accommodation**	**House Owner**	0.38	0.1-1.43		0.76	0.3-1.95	
	**Parents House**	0.72	0.37-1.41		0.86	0.55-1.37	
	**Rented House**	0.83	0.42-1.63		0.82	0.52-1.3	
	**Campus Accommodation**	1.00		0.47	1.00		0.85
**Smoking Status**	**Smoker**	1.01	0.65-1.59		0.91	0.63-1.32	
	**Non -smoker**	1.00		0.96	1.00		0.63
**Drug Use-Ever**	**Yes**	1.35	0.91-2.01		1.47	1.05-2.06	
	**No**	1.00		**0.14**	1.00		**0.02**
**Alcohol Use**	**Hazardous**	1.69	1.09-2.64		1.02	0.73-1.43	
	**Non-Hazardous**	1.00		**0.02**	1.00		0.89
**Physical Activity**	**Low**	1.00		**0.009**	1.00		0.76
	**Moderate**	0.49	0.31-0.78		1.11	0.77-1.62	
	**High**	0.61	0.37-1.02		0.97	0.63-1.49	
**BMI**	**Underweight**	1.00		**0.11**	1.00		0.4
	**Normal Weight**	1.65	0.38-7.22		1.33	0.7-2.51	
	**Overweight**	2.82	0.63-12.69		0.95	0.43-2.11	
	**Obese**	2.31	0.46-11.71		1.85	0.7-4.95	
**No. of sexual partners**	**None**	1.00		**0.02**	1.00		**0.009**
	**1-3**	2.25	1.21-4.18		2.04	1.22-3.42	
	**4-5**	2.79	1.29-6.06		1.8	0.95-3.4	
	**6 or more**	2.73	1.4-5.32		2.8	1.53-5.15	

Multivariate logistic regression, stratified by gender, was utilised to investigate the variables associated with positive mental health (above average mental well-being score). Controlling for age, there was an apparent inverse association between physical activity levels and the WEMWBS score among men. Men reporting both moderate and high levels of physical activity were significantly less likely to have an above average WEMWBS score relative to those with low physical activity. Similarly, men reporting 1-3, 4-5 or 6 or more sexual partners in their lifetime were significantly more likely to have above average mental health and well-being scores relative to those with no sexual partners. Among women, those with 1-3 or six or more sexual partners in their lifetime were over twice as likely to report above average mental health and well-being. However, no association between those reporting 4-5 sexual partners and mental health and well-being was observed. Results for multivariate logistic regression are presented in Table 4.

**Table 4:** Multivariate Logistic Regression: Risk factors associated with above average mental health and well-being (controlling for age).

	**Males**		
	**OR**	**95% CI**	**p-value**
**Physical Activity (IPAQ)**			
***Low***	1.00		**p=0.008**
***Moderate***	0.48	0.3-0.77	
***High***	0.59	0.35-0.99	
**No. of sexual partners**			
***None***	1.00		**p=0.004**
***1-3***	2.29	1.22-4.28	
***4-5***	2.98	1.32-6.72	
***6ormore***	3.64	1.78-7.43	
	**Females**		
**Year of Study**			
***1*^*st*^*year***	1.00		**p=0.03**
***2*^*nd*^*year***	0.7	0.46-1.06	
***3*^*rd*^*year***	0.75	0.46-1.21	
***4*^*th*^*year***	1.57	0.90-2.74	
**No. of sexual partners**			
***None***	1.00		**p=0.008**
***1-3***	2.18	1.28-3.72	
***4-5***	1.86	0.96-3.61	
***6ormore***	2.96	1.56-5.62	

* The initial model included: Age, BMI, physical activity, alcohol use, no. of sexual partners and drug use for men and age, year of study, drug use and number of sexual partners for females

## Discussion

This is one of the first studies to examine mental health and well-being scores in a broad, largely representative sample of third level students and the first to report on the factors associated with positive mental health and well-being using WEMWBS. The findings from this study suggest that students with a relatively adverse health and lifestyle profile have higher than average mental health and well-being. Significant, but relatively small differences in mental health and well-being scores were observed between men and women. However, no association between alcohol consumption, obesity, drug use or smoking status and positive mental health and well-being were detected in this research.

As highlighted by Cranford et al, the effect of alcohol use on mental health and well-being is unclear [15]. The current research did not uncover an association between alcohol use and positive mental health and well-being adding to the conflicting results of previous research. The Harvard college alcohol study found that poor mental health was associated with an increased risk of alcohol use. However, other research has reported positive associations between mental health and alcohol abuse with one study indicating “that associations between mental disorders and alcohol involvement among college students emerged only for alcohol dependence” [15].

When Needham et al investigated the effect of weight status on mental health and well-being among adolescents, the association between weight and mental health was context specific. Young girls and adolescents in lower grades experienced being overweight more negatively. Needham concludes that the rising rates of obesity among adolescents may act as a buffer to the stigma associated with being overweight and obese in this population [16]. This study found no association between BMI status and mental health and well-being in either men or women. This may be due to rates of overweight and obesity continuing to rise in Ireland. As more individuals become overweight stigma reduces, impacting less on the individuals mental health and well-being.

Research investigating the effect of smoking on mental health and well-being were more unified with strong associations between nicotine dependence and mood and anxiety disorders being evidenced [15]. In 2004, Patterson et al conducted a review of current research in the area concluding that symptoms of anxiety and depression are associated with cigarette smoking among American college students [17]. However, the current research is at odds with pre-existing knowledge in the area. No association between cigarette smoking and mental health and well-being in the current study was observed. Smoking habits are regularly underreported [18], an issue which may be skewing the results of this study.

Previous research investigating the effect of recreational drug use on mental health and well-being has provided conflicting results. In 2009, Cranford et al reviewed the literature surrounding mental health and well-being and recreational drug use [15]. This review provided differing results as to the relationship between recreational drug use and mental health and well-being. Some research reported a positive association between drug use and mental health and anxiety while others report a negative effect [15,19]. This study finds that recreational drug use is not associated with mental health and well-being among a third level student population.

Low levels of physical activity and one or more sexual partners in a lifetime were all associated with positive mental health and well-being in the current research. In 2005, Penedo undertook a review investigating the benefits of physical activity. This review identified a reduction in anxiety and depression among college students who completed physical activity [20]. Specifically, physical activity was associated with improved mood and a reduction in the symptoms of depression or anxiety. Several research studies have solidified this result [21–25]. This is, however, at odds with the current research where low levels of physical activity are associated with increased positive mental health and well-being among men. Levels of physical activity reported among our student population were relatively high, measured using the International Physical Activity Questionnaire (IPAQ). Despite the fact that the IPAQ has been extensively validated and recommended as an effective method of assessing physical activity participation [26], there are some limitations associated with its use. According to Saelens and Sallis (2003) one of the main limitations associated with IPAQ arises due to the fact that self-reported measures can often result in the over estimation of physical activity participation [27]. In fact, it has been suggested that the IPAQ may over estimate physical activity participation to a greater extent than other physical activity surveys [28,29]. Another limitation of the IPAQ is that survey participants can often experience difficulties distinguishing between moderate and vigorous intensity activities [30], a factor which can also result in the over estimation of physical activity. It is possible that these limitations affected the findings in our study.

In 2006, Martens et al reported on the differences between actual and perceived student norms in relation to sexual behaviour. Martens discovered that “students overestimated the sexual activity of the typical student” [31]. Sexual health is regularly viewed as a risk factor but is also a positive aspect of life. Some studies have reported high numbers of sexual partners as a normative occurrence among the third level student population [32,33]. Although risky behaviour, adherence to contraception and protection against sexually transmitted diseases should be of concern in this group, students may feel happier as they align themselves to the perceived sexual norms of their peers. Our finding of multiple lifetime sexual partners being associated with higher than average mental health supports this view.

### Strengths & Weaknesses

A strength of the current research is the rigorous sampling strategy utilised so that each degree programme, regardless of size, would have an equal opportunity of being included in the study. Also, student demographics from the overall institution where the study took place are similar to those sampled in relation to year in college, faculty and gender.

There are a number of potential sources of bias in cross-sectional studies including response bias and self-report bias, which may have affected the outcome of the study. Students who attend lectures may differ significantly from their non-attending peers in relation to their mental health and risk-taking behaviours, a concern which should be considered when interpreting results. When interpreting these results, the response rate of 51% should be taken into consideration. Though the response rate is moderate, it is in line with previous surveys undertaken both in Ireland [3] and abroad [34].

## Conclusion

The findings of this study are somewhat unusual in that students with an adverse lifestyle pattern have a higher than average mental health and well-being. Students represent a unique subgroup that is relatively privileged when compared to the general population [35]. Findings from this study may not be applicable to the general population. Previous research suggests that hazardous alcohol consumption and a high number of lifetime partners are social norms in this culture, meaning those closer aligned with the norms for their culture may report more positive mental health and well-being. This is evidenced in the current study. There is a need to replicate this research yielding a higher response rate to confirm the factors associated with positive mental health and well-being among third level students. A study of the general population is also warranted.
